# A pluralistic view of holobionts in the context of process ontology

**DOI:** 10.3389/fmicb.2022.911577

**Published:** 2022-08-04

**Authors:** Adrian Stencel, Dominika Wloch-Salamon

**Affiliations:** ^1^Institute of Philosophy, Jagiellonian University, Kraków, Poland; ^2^Faculty of Biology, Institute of Environmental Sciences, Jagiellonian University, Kraków, Poland

**Keywords:** symbiosis, unit of selection, hologenome, metaorganism, microbiome

## Abstract

Developing precise definitions and fine categories is an important part of the scientific endeavour, enabling fidelity of transfers of knowledge and the progress of science. Currently, as a result of research on symbiotic microorganisms, science has been flooded with discoveries which appear to undermine many commonly accepted concepts and to introduce new ones that often require updated conceptualisations. One question currently being debated concerns whether or not a holobiont can be considered an organism. Based on which concept, physiology or evolutionary, of the organism is chosen, the verdict differs. We attempt here to show how a change in perspective, from that of substance ontology into that of process ontology, is capable of reconciling opposing positions within the existing discussion and enabling the implementation of conceptual pluralism.

## Introduction

*Ontology* is the branch of philosophy concerned with what is and what sorts of things exist. It describes the attempt to devise a classification scheme that lists the underlying furniture of reality. Many classic questions in philosophy are, in fact, typical of the questions pursued by ontologists. For instance, does the world as we observe it really exist? Or is it perhaps some sort of projection of the human mind, such that, once the mind vanishes, the world does as well? Questions of this type are very common within philosophy (Bostrom, 2003), as well as in popular culture, as seen in the film *The Matrix*.

The main idea of *substance ontology*, one of the main traditions of thinking about ontology, is that the world constitutes of *things*, which are characterised by their properties (Robinson, 2021). For instance, Descartes thought that a substance is something that ‘depends on no other entities for its existence’. The essential property of a given substance, in this view, would be its independence of other substances (Morgan, 2021). Substance ontologists would say that in order to understand the structure of the world, one must understand *what sort of things exist* and *what kind of properties they have*. This ontological approach may seem well tailored to a biological way of thinking about the world, since an important part of biology is the discovery and description of new biological things and their properties.

Substance ontology is a broad idea within philosophy, encompassing at least two components with which most scholars, including biologists, would agree. These are the two main problems that substance ontologists are trying to solve. The first concerns *persistence over time.* If things exist in nature, they must possess some essential properties that are retained as time flows, enabling them to retain their identities over time. Even though some differences occur over a lifetime, a given individual is the same whether old or young, retaining some essential *substance* properties over time. Substance ontologists try to understand these properties. The second problem concerns the *question of boundaries*, i.e., how to define separate things in nature in cases where setting the boundaries between them entails potential problems. Famous problematic cases include foetus and mother (Kingma, 2019) and obligatory parasites vs. symbionts. If things truly exist, then there must always be a way to set boundaries between them. Understanding how to do this is thus part of the substance ontology research programme.

Biological sciences are well supplied with questions of an ontological nature. To *elucidate* questions about relationships and processes, it is necessary to determine what sorts of biological entities exist and what boundaries separate them. One way is to classify the studied world into fine biological categories, e.g., *species*. The clear classification of species is one of the field’s most widely debated issues (Ereshefsky, 1998; Pigliucci, 2003). The questions remain: What is the most appropriate definition of *species*?

Sorting things into fine categories with precise definitions is an important part of scientific endeavour. This effort is becoming one of the frontiers of biological interest, especially since science is being flooded with discoveries that appear to undermine many commonly accepted concepts. We believe that we are currently in the midst of this kind of change in biology due to the enormous impact of research on microbiology and the *microbiome* (see glossary; McFall-Ngai et al., 2013; Dittmer et al., 2016; Suárez and Stencel, 2020; Amato et al., 2021; Obrenovich and Reddy, 2022). This research is so important for biological theory that McFall-Ngai et al. (2013, p: 32–34) argued that ‘[t]hese new data are demanding a re-examination of the very concepts of what constitutes a genome, a population, an environment, and an organism’.

Understanding what an organism is, in the context of complex interactions between the host and its *microbiota* (see glossary), is at the forefront of this discussion. It has been shown that microbes perform a variety of functions and play vital roles in the functionality of hosts. Thus, perhaps a host and all of its symbiotic microbes, collectively called a *holobiont* (see glossary), should be considered a genuine individual. Some researchers (Zilber-Rosenberg and Rosenberg, 2008; Gilbert et al., 2012; Doolittle and Booth, 2017; Suárez, 2020) promote this approach, while others are far more sceptical (Douglas and Werren, 2016; Skillings, 2016; Stencel and Wloch-Salamon, 2018).

The status of the holobiont as an organism depends on which concept of the organism has been adopted, as discussed in the literature (Gilbert et al., 2012; Pradeu, 2016b; Stencel and Proszewska, 2018). In this paper, we wish to further support this view by showing that process ontology can, along with conceptual pluralism, offer an interesting justification regarding holobionts. In the following section, we will attempt to reconcile the physiological and evolutionary approaches to holobiont individuality within the framework of process ontology. We present our arguments from the perspective of hosts. However, the perspective of the microorganisms could be assumed as well (see Suárez and Stencel, 2020).

## Process ontology

The concept of process ontology can be traced back to an ancient Greek philosopher known as Heraclitus (535–ca 475 bc), who believed that the foundation of reality is change, and that *things* exist only temporarily. He is well known for the phrase *panta rei* (‘everything flows’). Throughout the history of Western philosophy, there have been attempts to develop the heritage of Heraclitus. The most famous advocates were Alfred North Whitehead, Martin Heidegger, and Gottfried Wilhelm Leibniz, who relied on a processual way of thinking about the world (for a review of the history of process ontology, see Seibt, 2022). The philosophy of biology in the twentieth century developed without a strong connection to this concept.

However, currently, process ontology is undergoing a renaissance in the philosophy of biology, as scholars are beginning to think that it is a more appropriate framework to think about and to describe living objects (Meincke, 2018; DiFrisco and Jaeger, 2019). For instance, Nicholson and Dupré (2018, p: 2) wrote:

[…] the living world is a hierarchy of processes, stabilised and actively maintained at different timescales. We can think of this hierarchy in broadly mereological terms (see glossary): molecules, cells, organs, organisms, populations, and so on. Although the members of this hierarchy are usually thought of as things, we contend that they are more appropriately understood as processes.

*Process ontology* focuses on the idea that the foundations of reality are not made of *things*, but of constantly changing *processes*. Such processes are not characterised by any essential, constant properties. The properties of this kind of process change, with old ones disappearing and new ones emerging, although they may attain a temporal stability and appear to us as ‘things’, as discussed below. Nothing is constant except change, and as long as the processes persist they will undergo dynamic change. This view of reality has certain consequences, two of which are very important. The first concerns identity over time. Processes retain their identity over time not because they have certain properties, but because they persist; the longer they persist, the more they change. As a result, they may have very different (in terms of properties) temporal parts. Only when we combine all those temporal parts will we see the whole. In other words, processes have both temporal and spatial parts. The latter concern the question of boundaries. If processes are dynamic and undergo constant change, we should not expect any clear boundaries between different processes or even between the parts of a given process. A good biological example is pregnancy, where it is problematic to set boundaries between a mother and her child, especially at the early stages of development (Kingma, 2019).

Process ontology does not rule out the existence of *things*; it simply rejects the idea that *things* are the building blocks of reality. Instead it is *processes* that constitute these blocks. These processes are complex and dynamic, intertwined and interdependent. Processes produce many different kinds of emergent phenomena, *things*, which are ephemeral. Some appear as cohesive wholes long enough to be perceived by humans. Such temporal, static, and cohesive manifestations of dynamic processes constitute *things* in process ontology. In other words, things are derivative, and processes are fundamental, as expressed by Nicholson and Dupré (2018, p: 11):

Instead of thinking of processes as belonging to things, we should think of things as being derived from processes. This does not mean that things do not exist, even less that thing-concepts cannot be extremely useful or illuminating. What it does imply is that things cannot be regarded as the basic building blocks of reality. What we identify as things are no more than transient patterns of stability in the surrounding flux, temporary eddies in the continuous flow of process.

## The holobiont concept of the organism

**Figure 1:**
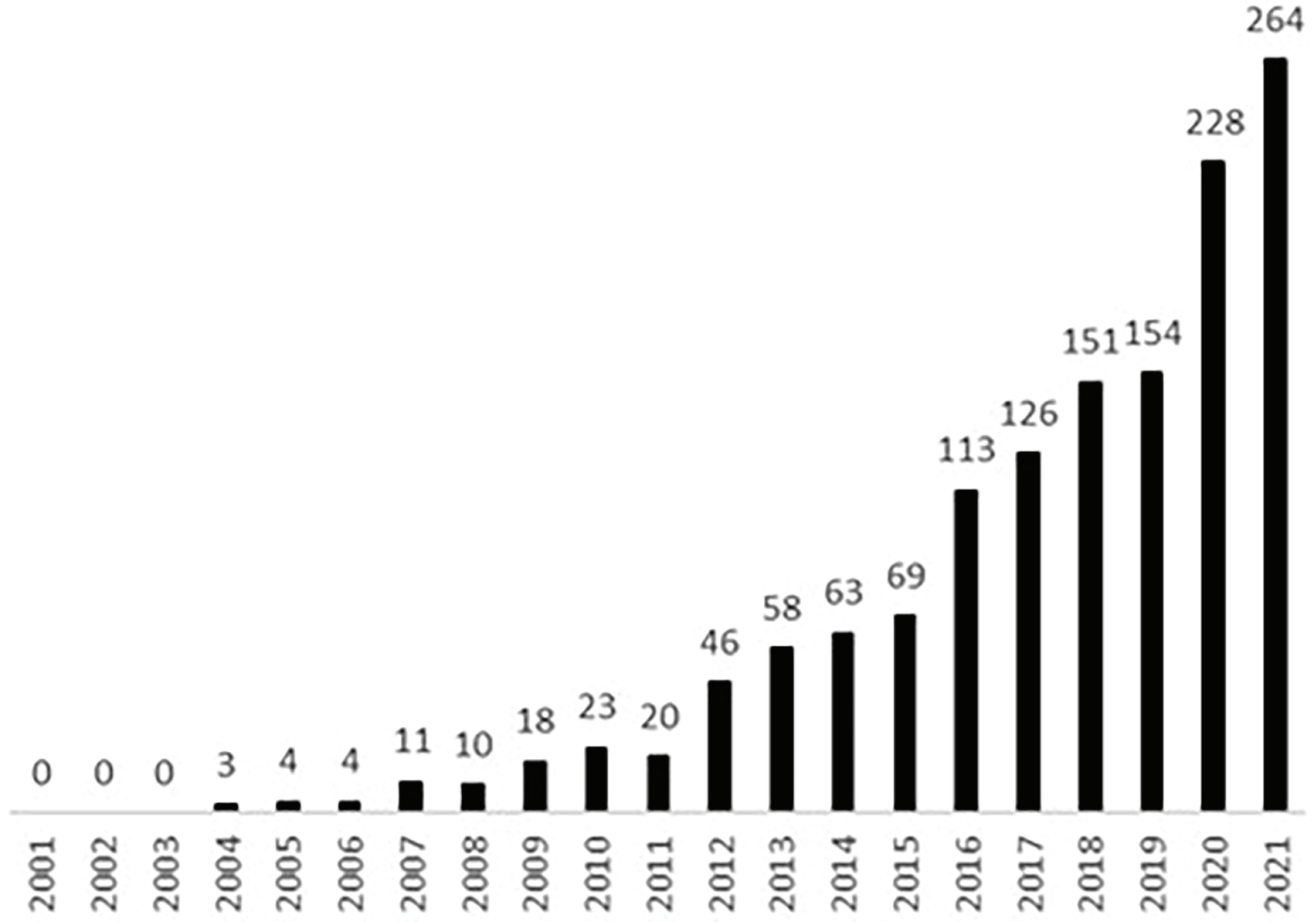
Number of publications listed in Scopus that include the term holobiont in the title, abstract, or keywords within the period 2001–2021, (entry dated 1 June 2022).

The term *holobiont* was introduced by Margulis (1991) to describe a host along with its obligatory symbiotic microbes. The meaning was subsequently expanded to encompass a host along with all of its symbiotic microorganisms (Zilber-Rosenberg and Rosenberg, 2008). In recent years the use of the word *holobiont* in scientific papers has become increasingly popular (see Figure 1). This has prompted questions about the meaning of the term. The most important question seems to be: are holobionts genuine organisms or not? It turns out that, due to the plurality of the concepts of organisms, the answer to this question ultimately depends on the concept of organism in use. In the next two sub-sections we will show this by demonstrating that each selected physiological and evolutionary concept of the organism renders a different verdict about the organismal nature of holobionts.

### The physiological view of organisms and holobionts

The crucial part of physiology is an attempt to answer the proximate questions, starting with ‘how?’, and to identify the mechanisms that keep organisms cohesive and functional over time. This has led to the *physiological concept of the organism*. For instance, Gilbert et al. (2012, p: 329) stated that ‘[…] *the physiological* view of animal individuality regards the organism as comprised of parts that co-operate. Similarly, Godfrey-Smith (2013, p: 12) wrote that, from the metabolic perspective, ‘[o]rganisms are systems comprised of diverse parts which work together to maintain the system’s structure, despite turnover of material, by making use of sources of energy and other resources from their environment’. This leads to the question: do symbiotic microorganisms play such a role in the host’s physiology? Should a holobiont be considered a physiological organism? Three arguments, namely (i) activity, (ii) spatiotemporal position, and (iii) tolerance of the immune system, support the physiological concept of the holobiont as a genuine organism (Bordenstein and Theis, 2015; Stencel and Proszewska, 2018; Triviño and Suárez, 2020; Boem et al., 2021).

Firstly, the microbiome, like the host’s cells, actively participates in building and maintaining the host’s body. These microbes process and assimilate external resources and thus contribute to maintaining the viability of the whole organism over time. For example, the digestion of certain food components such as cellulose can be carried out in the termite gut only with the help of suitable microorganisms (Scharf and Peterson, 2021). Thus microbiomes are not simply elements of the environment, processed and assimilated by the host’s cells in the same way as oxygen, water, or other substances (Stencel and Proszewska, 2018; Triviño and Suárez, 2020).

Secondly, one very specific fact about symbiotic cells is that they can be found within the host’s body and are thus able to participate in the network of interactions of host cells. Due to their size, symbiotic cells can occupy a specific space within the host’s body (Suárez and Stencel, 2020). As such, they can be embedded within the host’s biochemical cycles in a very subtle way. For instance, in the process of digestion in humans, the host’s cells accomplish the preliminary stages, whereas the microbes take over the subsequent steps (Larsbrink et al., 2014). Furthermore, these interactions are not constrained to a single point in time, but can take place throughout the host’s entire life. Some symbiotic microorganisms are present within a host from its birth to its death. Of course, their taxonomic and functional composition changes throughout the host’s lifetime (Reveles et al., 2019), but the host constantly interacts with and depends on some microbial cells.

The third argument is based on the immunological criterion (Pradeu and Carosella, 2006; Pradeu, 2016a). The immunological system is known to be an important part of the physiology of animals, providing a defence against pathogens. The immune system constitutes a discrimination mechanism that accepts some entities while rejecting others. The line of discrimination lies between the self and the non-self (Burnet, 1962). In other words, the self/non-self theory states that the immune system makes no response to endogenous components of the organism (‘self’-like host cells), while rejecting exogenous constituents (‘non-self’-like viruses or cells of other organisms). The problem with the physiological definition of the organism is that many genetically foreign entities may be accepted by the immune system, while many endogenous entities are routinely destroyed. Currently, the *discontinuity theory of immunity*, which proposes that immune responses are triggered by sudden changes in the molecular motifs that interact with the receptors of the immune system, is considered more convincing (Pradeu and Carosella, 2006; Pradeu, 2020). Thus, microbes which do not trigger this kind of immune system are tolerated. As such, they are treated as genuine parts of the physiological individual. Of course, tolerance does not mean simply presence within the body of the host, as some pathogenic microbes can achieve this by penetrating the immune system. Tolerance is rather a complex and intimate dialogue between the host’s immune system and microbes, one that allows the entry of the latter into the former (for details, see Pradeu, 2020).

### The evolutionary view of holobionts

From the evolutionary perspective, a host and its microorganisms may be considered an evolutionary organism constituting a *unit of selection* (Zilber-Rosenberg and Rosenberg, 2008; Gilbert et al., 2012; Bordenstein and Theis, 2015; Doolittle and Booth, 2017; Lloyd and Wade, 2019; Suárez, 2020)—but only if holobionts fulfil the criteria of reproduction and heredity, variance, and fitness differences (Godfrey-Smith, 2013; Skillings, 2016) they are considered eligible to be units of selection (see glossary). We will argue, through a discussion of their hereditary nature, that in most cases this is very unlikely.

Heredity implies that there is similarity in the parent-offspring lineage. Parents that reproduce are capable of passing on their genes to their offspring. Reproduction can take different forms, however (Godfrey-Smith, 2009). One example of a simple reproducer, i.e., one that requires only outside resources to reproduce, might be a bacterial cell. Another category comprises scaffolded reproducers. These are units whose reproduction depends on the existence of other reproducers. A good example of this category might be viruses that require the use of the host’s biochemical machinery to reproduce. A collective reproducer is made up of units that can reproduce themselves (that is, of simple reproducers). A classic example is a multicellular organism that can reproduce itself but that is made up of cells.

The transmission of microbiota and the relative significance of vertical *vs* horizontal transmission can be measured as a quantitative parameter, such as *transmission fidelity* (see glossary), which may be very low and acquired chiefly through the environment, in which case parents and their offspring may have different microbiomes. At the other extreme, transmission fidelity may be very high, with strict vertical transmission; in such a case, the microbiomes of parents and their offspring would be very similar. Therefore, if a host has microbiome with a high level of transmission fidelity, then such a combination would form a unit of selection. Similarly, we have suggested that only a fraction of microorganisms can function along with a host as a unit of selection that influences the variation and fitness of the individual (Stencel and Wloch-Salamon, 2018). In particular, a model based on multi-level selection theory encompassing mutations and variations within the microbiota identifies conditions important for the selection for the holobiont as an evolutionary entity (van Vliet and Doebeli, 2019). In this case the vertical transmission of the microbiota prevails over horizontal, and their decay, in conjunction with the short time required for the generation of the host, is slow. The authors have suggested that these conditions could be fulfilled in certain insect or other short-lived species but are unlikely to occur in long-lived mammals.

A good example is the case of the symbiotic bacteria *Buchnera* sp. and their multicellular hosts, which form a very ‘tight’ relationship (Chong et al., 2019). Members of *Buchnera* sp. have lost many necessary genes in the course of evolution. As a result, they are not capable of performing many vital functions and therefore cannot return to a free-living state; they have to live within the bodies of hosts. This is beneficial for aphids, as these microorganisms provide the necessary nutrients that are lacking in the aphids’ diet (essential amino acids). Thus, to ensure the presence of these beneficial microbes in every succeeding generation, aphids transfer them to their offspring *via* special propagules. These microbes and aphids, due to their co-evolution, constitute a reproducer.

The majority of symbiotic microorganisms is transmitted horizontally (see Douglas and Werren, 2016; Skillings, 2016; Suárez and Stencel, 2020). For instance, the human gut microbiome is very important in terms of contributing to digestion (Larsbrink et al., 2014), but the majority of microbes that reside there are acquired from the environment in various ways (e.g., Dill-McFarland et al., 2019). Therefore, the majority of the holobionts that have been described so far cannot be classified as units of selection, if vertical transmission is necessary requirement as we argued. Note that the majority of gut microbiota would be considered components of physiological individuality due to their enormous impact on the host. This clearly shows that the two concepts render different verdicts concerning holobiont.

## Pluralism concerning holobionts in the context of process ontologies

The last section showed that whether holobionts are organisms or not ultimately depends on the concept of the organism in use. For instance, a positive answer is not always appropriate in the case of an evolutionary organism, due to the absence of vertical inheritance in the majority of holobiont cases. The physiological organism concept appears more conducive to consideration of holobionts as organisms due to the deep involvement of symbiotic microorganisms in the maintenance of hosts’ functionality. How can we justify the existence of this pluralism?

The most promising way is to place the question within the context of pragmatism, i.e., a philosophy that emphasises the practical consequences of theories rather than their accuracy in describing the world. Pragmatic philosophy offers its own interpretation of nearly every concept discussed in philosophy (Legg and Hookway, 2021; Proszewska, 2022). In this spirit, given that scientists have differing research aims, each of which is unique, requiring a specific conceptual framework, recent philosophical approaches assume that there is a place for multiple concepts of the organism (Pepper and Herron, 2008; Kovaka, 2015; Stencel and Proszewska, 2018). Thus, for different research aims, researchers construct different concepts of the organism that simply fit their needs more closely. As a result, the concepts that are used to study the interactions of hosts and microbes are pragmatic. Rather than representing the world ‘as it is’, they are conceptual tools created by scientists in order to pursue their scientific goals (Kovaka, 2015; Stencel and Proszewska, 2018). However, since scientists tend to believe that their research is an attempt to unravel the very nature of reality, as opposed to the creation of ‘fictional tools’ for pragmatic reasons, this justification of pluralism might raise objections and night not necessarily be welcomed. Sagan (1995, p: 27) once wrote:

The truth may be puzzling. It may take some work to grapple with. It may be counterintuitive. It may contradict deeply held prejudices. It may not be consonant with what we desperately want to be true. But our preferences do not determine what’s true. We have a method, and that method helps us to reach not absolute truth, only asymptotic approaches to the truth – never there, just closer and closer, always finding vast new oceans of undiscovered possibilities.

**Figure 2:**
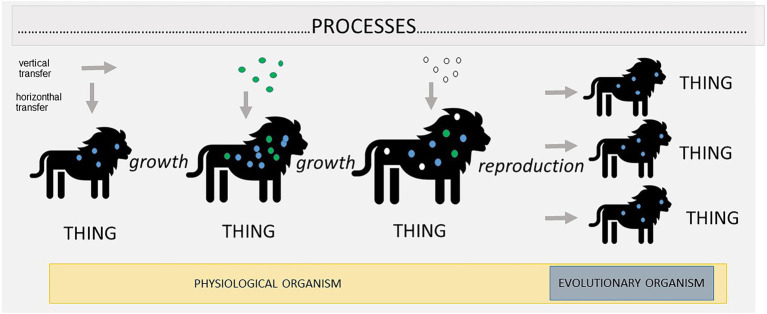
This represents the idea that holobionts are processes. The nature of processes is constant change, captured here by the growth of the host and change in the composition of its microbiota. Constant change does not imply that there is no stability at all. Processes may become sometimes so intertwined that they appear as cohesive wholes, called “things.” We believe that, at least in the case of holobionts, two such structures emerge: physiological individuality, in which cohesion is obtained through physiological interactions, and evolutionary individuality, in which cohesion is obtained through vertical transmission of microbes.

We think that the concept of process ontology can provide a better understanding of this pluralism in biology, as well as a suitable justification for its own existence which is, moreover, congruent with the spirit of scientific investigations. Process ontology, as stated earlier, assumes that at the foundation of reality there are processes that undergo constant change. Furthermore, the longer processes persist, the more they may change. Organisms in such an ontology would also be seen as processes (albeit quite complex ones, interconnected to some extent due to their evolutionary history) that undergo constant dynamic changes. Different parts of this process may differ from each other substantially, just as different spatial parts of an organism differ—we might think of a lion (Figure 2). As a result, in this kind of ontology, organisms have not only spatial but also temporal parts. A surprisingly similar view was expressed by Woodger (1929) in his book *Biological Principles* almost 100 years ago (p: 299):

An organism, whatever else it may be, is an event – something happening. It is temporally as well as spatially extended. It has temporal as well as spatial parts. Your pet dog today and your pet dog yesterday are two different temporal parts of the same dog, just as his head and his tail are two different spatial parts of the same dog. It is in virtue of the particular kind of continuity of the dog yesterday and the dog today that we call it the ‘same’, and this seems to be the proper sense of the term. But it can no more be taken for granted that today’s temporal part is the same as yesterday’s than it can be taken for granted that one spatial part, e.g. the head, is the same as another, e.g. the tail. We know, in fact, that they are not the same. Organisms are temporally as well as spatially differentiated.

This way of thinking about organisms justifies pluralism. Mainly, different concepts of the organism emphasise different properties as necessary in order to consider something an organism. At first glance, these concepts seem to conflict with each other, as they favour different properties as being crucial in this regard. However, if we combine this with the idea that organisms are processes, the contradiction disappears. Simply understood, different concepts of the organism capture different parts of processes characterised by different properties. Therefore, these concepts focus not on the organism as a whole, but on its temporal parts! As result, it is only when we combine all these concepts that we capture the organism as a whole. In other words, we capture the processes of being an organism.

But why do those parts, captured by different concepts, sometimes look like organisms? Why do they look like cohesive wholes with clear boundaries? For instance, if we observe a cat on the fence, it appears a certain way. Process ontology explains this. Sometimes processes become so intertwined and connected that they become a ‘thing’—a temporal and cohesive manifestation, formed as the nexus of many intertwined processes. The concept of a ‘thing’ thus understood completes the process ontology in question and enriches our justification of pluralism. Organisms are specific biological processes interconnected to the extent that they manifest themselves in the form of a thing. Notably, as the process undergoes constant changes, they may, at various points of their persistence, manifest themselves as different things. Concepts of the organism represent these various temporal manifestations.

This all provides a good explanation for the pluralistic view of holobionts (Figure 2). Holobionts are very complex processes. Some aspects of these processes become so integrated and intertwined that they become a ‘thing’. Mainly, they form physiological organisms—a temporal thing that independently retains its structure over time. This happens because of the work of its somatic cells and because of the interactions with microbes (acquired mainly horizontally) that are incorporated into and that begin to serve a certain role within its biochemical network. The same, however, cannot be said about evolutionary individuality, because, as stated earlier, the majority of the microbes that play an important physiological role are not inherited vertically. As a result, they are not part of the evolutionary individual. Overall, processes within a holobiont are extremely complex and dynamic, and, as they persist and undergo changes, a different ‘thing’ emerges from these processes. Sometimes all of these processes become intertwined, as in the case of physiological individuality, and sometimes only a small fraction, as in the case of evolutionary individuality. This explains why some scholars treat a holobiont as an organism and others do not. They are simply focusing on different temporal parts of the processes in question.

All of this may suggest that we defend an anarchistic approach to the problem of holobionts, namely, that any concept one chooses is justified. While we certainly are sympathetic to a pluralistic approach, we do not wish to go too far. We believe that there is a place for pluralism, which is appropriate, as we show, when scientists approach different parts of the processes—as in the case of the evolutionary vs. physiological approaches presented in this paper. At the same time, we believe that there is a place for healthy argument and discussion, which is appropriate when scholars are examining the same part of the processes. The debate about the evolutionary status of holobionts is a good example: the disagreement concerns whether horizontally transferred microbes (e.g., Kikuchi et al., 2007), or microbes with a *mixed mode of transmission* (e.g., Russell, 2019) can be considered components of an evolutionary organism (see for a discussion Zilber-Rosenberg and Rosenberg, 2008; Bordenstein and Theis, 2015; Theis et al., 2016; Lloyd and Wade, 2019; Suárez, 2020). Indeed, scholars may disagree about the applicability of a given concept (e.g., in the case of different evolutionary approaches) in capturing a certain part of processes, but may nevertheless embrace pluralism as long as the concepts refer to different parts of the processes. Therefore, our approach does not minimise the complexity of such debates. Rather, we assert that, as long as different concepts capture different parts of the processes, there is room for reconciliation.

## Concluding remarks

In this paper, we have attempted to justify the existing pluralistic approach to defining organisms and holobionts. We have shown how changing an unconscious approach involving *substance ontology* into an approach involving *process ontology* can contribute to the existing discussion about pluralism concerning holobionts. Our underlying agenda was to show that some views that appear inconsistent can be reconciled if we place them in the context of larger philosophical frameworks. We hope this will attract biologists to regard some philosophical ideas more sympathetically in their future considerations. Furthermore, we hope that we will attract more philosophers to biology, which, due to its complexity and diversity, is a fantastic arena for the implementation of different philosophical ideas. There are still many interesting questions concerning process ontology that can be explored by biologists and philosophers alike. We believe that such recombinations of ideas can influence biological theories and hypothesis and thus contribute to the advancement of science (Box 1).

Box 1. **Outstanding questions for further investigations**

1. According to the concept of process ontology, boundaries between processes are fuzzy. If processes are fuzzy, we can argue that there is only one process that encompasses all of the species, ecosystems, etc., which constitute temporal manifestations thereof. This is a radical interpretation of process ontology (Morgan, 2021). If valid, this would provide arguments in favour of the Gaia hypothesis (e.g., Lenton et al., 2018), which states that the earth is one big organism. In this case, the Earth, would represent a single process.
2. A thing is a coherent manifestation of a process. If a holobiont is composed of processes, then those things are represented by different concepts of the organism (Figure 2). In this paper we have discussed the evolutionary and physiological concepts of the organism. However, as Clarke (2010) argued, many more concepts of the organism are in circulation. Perhaps some of them should be considered as representations of temporal parts of the holobiont.
3. If things are cohesive manifestations, then what sort of cohesiveness should a process obtain in order to manifest a thing? Is it possible to quantify this cohesiveness?
4. If the concept of process ontology is supposed to be useful for biology, it must be universally useful. What are other areas of biological investigation to which it can be applied? Are there any other problems that can be solved by shifting to a processual view of reality?
5. In this paper we took a perspective centred on hosts, but a microbe-centric perspective is as well a justified position, worthy of consideration (Suárez and Stencel, 2020). This raises a question: if we alter our perspective in this way, can we consider a holobiont to be a ‘thing’ from the viewpoint of microbes?
6. Pluralism concerning holobionts encompasses concepts that focus on different parts of processes. However, scholars can (and should) disagree about the applicability of a given concept. We defended a view that limits the status of an evolutionary organism to a host and vertically inherited microbes. There is, however, ongoing debate about whether this concept should not be expanded to include at least some horizontally acquired micro-organisms. More concerning this issue can be found in the literature (Zilber-Rosenberg and Rosenberg, 2008; Bordenstein and Theis, 2015; Lloyd and Wade, 2019; Suárez, 2020).
7. In this paper, we discussed whether a holobiont can be a certain type of organism. If we agree that a holobiont is an evolutionary or physiological individual unit of selection, this raises a question: how could such a thing have evolved in the first place? This very important question is currently being widely discussed in relation to holobionts (Bordenstein and Theis, 2015; Theis et al., 2016; Lloyd and Wade, 2019; Suárez, 2020) and organisms in general (Maynard-Smith and Szathmary, 1995; Okasha, 2006; Godfrey-Smith, 2009; Opalek and Wloch-Salamon, 2020).

## Data availability statement

The original contributions presented in the study are included in the article/supplementary material; further inquiries can be directed to the corresponding authors.

## Author contributions

AS and DW-S contributed equally to conceptualizing and writing the manuscript. All authors contributed to the article and approved the submitted version.

## Funding

The work was funded by the National Science Centre, Poland *via* an OPUS grant to DW-S. No. 2017/25/B/NZ8/01035, and the Biology Department Research subsidy No. N18/DBS/000019 and by Research Non-Fungible Token (rNFT) “The Ontology of the Holobiont” to AS and Biology Department research subsidies N18/DBS/000003.

## Conflict of interest

The authors declare that the research was conducted in the absence of any commercial or financial relationships that could be construed as a potential conflict of interest.

## Publisher’s note

All claims expressed in this article are solely those of the authors and do not necessarily represent those of their affiliated organizations, or those of the publisher, the editors and the reviewers. Any product that may be evaluated in this article, or claim that may be made by its manufacturer, is not guaranteed or endorsed by the publisher.

## Glossary

Evolutionary concept of the organism—definition of an organism as a unit that undergoes evolution by natural selection as a whole. We consider such an organism synonymous with a unit of selection.

Holobiont—an aggregation of the host and all of its symbiotic microorganisms.

Horizontal transmission—the transmission of micro-organisms between members of an ecosystem that are not in a parent-progeny relationship.

Microbiome—a community of microorganisms which can be found either within/on a multicellular host or outside it, e.g., a soil microbiome.

Mixed Transmission—incorporates some rate of *horizontal* and vertical *transmission.*

Mereology—the philosophical theory of parthood relations: of the relations of a part to the whole and the relations of a part to a part within a whole.

Microbiota—a community of microorganisms that can be found in or on a multicellular host.

Obligatory symbiosis—a type of symbiosis where one or both of the symbionts entirely depends on the other for survival.

Ontology—a branch of philosophy that studies the structure of reality at the most fundamental level. The main question is *what exists?*

Process ontology—a tradition of thinking about reality, and a branch of philosophy which emphasises that there are processes at the foundations of reality that constantly change. Things are not fundamental; rather, they are derived from processes; they are temporal, cohesive manifestations of those processes.

Physiological concept of the organism—definition of organisms as systems of elements that co-exist, thus sustaining the structure and functionality of a whole.

Pragmatism—a broad tradition of thinking about reality emphasising the need to focus on the practical consequences of concepts/theories, which are merely tools used to investigate the world. The degree of accuracy achieved in describing reality ‘as it is’ should not be a matter of concern.

Reproducer—any entity capable of reproduction. In Godfrey-Smith’s framework, a reproducer is a unit of selection.

Substance ontology—a broad tradition of thinking about reality, emphasising that there are certain things with certain properties at the foundations of reality. Things are fundamental elements of reality.

Transmission fidelity—the relative significance of vertical compared to horizontal transmission.

Vertical transmission—the transfer of microbial symbionts from parent/parents directly to offspring.
